# The Long-Term Observation of the Beneficial Effects of Treatment: 0.12 mg Anti-VEGF Monotherapy or Anti-VEGF Combined Therapy and Diode-Laser in Various Stages of Retinopathy of Prematurity—Series of Cases

**DOI:** 10.3390/jcm12175644

**Published:** 2023-08-30

**Authors:** Monika Modrzejewska, Martyna Nazwalska

**Affiliations:** 1 Second Department of Ophthalmology, Pomeranian Medical University in Szczecin, Powstańców Wielkopolskich 72, 70-111 Szczecin, Poland; 2 Department of Ophthalmology, Independent Public Health Care Center of the Ministry of the Interior and Administration (SP ZOZ MSWiA), ul. Jagiellońska 44, 70-382 Szczecin, Poland; marting8@wp.pl

**Keywords:** retinopathy of prematurity, anti-VEGF, diode laser, beneficial effects of therapy, ROP complications

## Abstract

Background 2-year observations of ranibizumab monotherapy and combined therapy with diode laser for severe ROP in extremely prematures. Materials and methods: In a group of 18 prematures (*n* = 36 eyes; 5 study groups); 25.8 ± 1.5 Hbd, birth weight 796.5 ± 166.1 g. Apgar 4.62 ± 1.88) with A-ROP (n = 22; 61%) and 3 ROP (plus) (n = 14; 39%), ranibizumab monotherapy (n = 4 eyes) in dose 0.12 mg/0.12 mL or with diode laser (n = 32 eyes) were applied. The first intervention was carried out in PMA of 33 (gr. 4 and 5) and 34 in (gr. 1, 2, 3), mean follow-up time 21.44 ± 8.7 months. One-way analysis of variance (ANOVA) with Welch’s correction, non-parametric Kruskal-Wallis test, Chi square test of independence were used. A retrospective observational study based on a case series. Results Retinal attachment was achieved in 92.3% of the studied eyes. Bilateral retinal detachment was noted in 1 infant (2 eyes). Myopization (−0.75 to −7.5 D) was observed in 5 infants (45%); mild hyperopia (+0.5 to +4.5 D) was observed in the rest infants (55%). Conclusions Individualization strategies in severe ROP with lower dose 0.12 mg Ranibizumab or combined laser-therapy resulted in effective outcomes. Myopia has not been reported in patients where Ranibizumab was the first drug administered in the ROP treatment strategy.

## 1. Introduction

Retinopathy of prematurity (ROP) is a vasoproliferative retinal disease where abnormal vessels develop. It occurs only in premature infants and remains the leading cause of blindness in infants in general (3–10% worldwide) [1,2]. Currently, the main two sources of origin are considered to be low-risk ROP of the body and the extreme immaturity of the child. A multicenter study on cryotherapy in ROP (CRYO-ROP, The Multicenter Trial of Cryotherapy, Retinopathy of Prematurity Study) involving 4099 babies with birth weight ≤1251 g baby that birth weight and early gestational age were strong due to the development of the “threshold” ROP [3]. The most frequently identified characteristic factors of the form of ROP have been developed based on the definition in the neonatal period and Western mechanical ventilation. Relative hypoxia during fetal life stimulates the development of the retina by affecting secretion hypoxia-inducible factor 1 (HIF-1), which regulates vascular endothelial growth factor, VEGF. VEGF is mainly secreted by retinal astrocytes and Muller cells and subsequently stimulates the differentiation, proliferation and migration of vascular endothelial cells volleyball [4]. HIF-1 is rapidly degraded under normoxic conditions, but in fetal life, during which conditions of moderate hypoxia occur, its half-life is prolonged and results in its accumulation. The development of retinal vessels consists of two phases: vasculogenesis and angiogenesis. The first phase is characterized by de novo formation of blood vessels from endothelial precursor cells in the central part of the retina, while the second phase is the development of blood vessels, which originate in vessels that already exist [5]. This occurs during the period of vasculogenesis to create four vascular arcades. It is during angiogenesis that vessel density increases.

In preterm birth, the developing retina is exposed to conditions of relative hyperoxia, which are often further enhanced by supplemental oxygen therapy. This results in a decrease in the level of HIF-1, and consequently a reduction of factors that stimulate the physiological maturation of retinal vessels, i.e., VEGF and insulin-like growth factor 1 (IGF-1). Obliteration of blood vessels takes place and, subsequently, a sudden inhibition of growth retinal vessels occurs—the first phase of ROP. The second phase begins between 31 to 34 weeks of postpartum life and is characterized by uncontrolled vascular growth of the retina induced by relative hypoxia. This process is caused by numerous growth factors secreted by the peripheral ischemic, non-vascularized retina such as VEGF, IGF-1, Transforming growth factor beta 3 (TGF-β3), erythropoietin (EPO), angiopoietin-like protein 4 (ANGPTL4) and HIF1α [6,7,8,9]. New abnormal vessels are created at the border of the vascularized and nonvascularized retina. These vessels then penetrate into the vitreous body, and if this process is not inhibited, they develop further leading to hemorrhage and tractional retinal detachment responsible for the development of abnormal retinal vasculature and subsequent complications such as retinal detachment and long-term vision impairment [10].

At present, laser photocoagulation of the uninvolved retina, based on recommendations from the ETROP (Early Treatment for Retinopathy of Prematurity) study in 2004, is still the standard treatment for ROP. This approach has been modified by the BEAT-ROP (Bevacizumab Eliminates the Angiogenic Threat—Retinopathy of Prematurity) study of 2011, which was the first randomised clinical trial to evaluate the efficacy of intravitreal monotherapy with an anti-VEGF drug (Bevacizumab) in the treatment of ROP. The RAINBOW trial of 2019, which evaluated the efficacy and safety of the anti-VEGF intravitreal antibody Ranibizumab [11], with a shorter half-life and potentially lower systemic toxicity than bevacizumab, [12] (7.19 and 9.82 [13] subsequent days) was groundbreaking for the treatment of ROP. In 2009, Ranibizumab (Lucentis, Novartis) was approved by the European Medicines Agency as the only drug for the treatment of severe forms of ROP. According to product characteristics, this drug at a dose of 0.2 mg/0.02 mL is suggested in ROP for premature infants with birth weight <1500 g with ROP in zone I (stage 1+, 2+, 3 or 3+), zone II (stage 3+) or in Aggressive ROP (A-ROP) [14].

Taking into consideration the incessant worldwide interest in the effects of treatment of severe ROP, especially with the use of anti-VEGF substances, coupled with a lack of uniform criteria in the literature for choosing the order of procedures—monotherapy or combined therapy—the authors of this article present their own long-term observations (mean 21.44 ± 8.69 months) of therapy conducted in various severe forms of ROP.

The authors present selected cases of extremely low-weight infants and analyze the long-term effects of the applied treatment in monotherapy (diode-laser or intravitreally Ranibizumab IVR) as well as in combined therapy (diode–laser and Ranibizumab) administered in different order in severe forms of the disease, i.e., A-ROP and stage 3 with plus disease at a reduced dose of 0.12 mg.

## 2. Materials and Methods

A retrospective observational analysis of the ocular condition in a case series based on a clinical retinal examination using RetCam 3 (Consultronix) was performed in a group of 35 eyes with severe ROP in 18 premature infants (3 females–16.7% and 15 males–83.3%) treated with anti-VEGF monotherapy or combination therapy: intravitreal anti-VEGF and diode laser or diode laser and successively anti-VEGF in different coincidence–due to severe and advanced ROP–comorbidities and demographic characteristics with a type of severe of ROP are presented in Table 1 and Table 2 respectively. Indications for treatment with a diode ROP laser included: any stage of ROP in zone I with the “plus” symptom Stage 3 without the “plus” sign in zone I Stage 2 or 3 with a plus sign in zone II. (W tym zdaniu chyba brakuje ze 3 przecinki—nie wiem ktore stadium, ktora zona) The plus sign should involve at least two quadrants.

A different management strategy was used in the treatment of advanced ROP or A-ROP in a group of extremely low-weight premature infants. At different follow-up dates, subsequent ophthalmological controls observed, features of regression of retinal vasculature lesions, with a focus on the observation of the therapies used, indicating whether one of the therapies used has an advantage in efficacy over the others. Observations were conducted with the RetCam3 ophthalmic tool. The study received a positive opinion of the Bioethics Committee at the Pomeranian Medical University (Consent, Nr KB-0012/132/16). The study protocol was compliant with the requirements of the Helsinki Declaration. Prior to the study, written consent was received from each from each child’s guardian. The patients were informed about possible complications related to the examination as well as clinical, surgical and therapeutic procedures.

Premature infants with ROP were divided into 5 groups based on the employed management strategy, which included monotherapy, diode-laser or anti-VEGF or combined therapy (anti-VEGF, laser or laser and anti-VEGF simultaneously or sequentially) with different intervals between courses of therapy. Intravitreal ranibizumab was administrated in doses of 0.25 mg/0.025 mL (23 eyes) or 0.12 mg/0.12 mL (12 eyes). It should be noted that half of the anti-VEGF drug/Ranibizumab/dose 0.12 mg was used relative to the manufacturer’s recommendation according to Stahl, A. et al. [15]. Characteristics of coexisting diseases in the study group are presented in Table 1.

Additionally, in the study groups, refraction after previous cycloplegia (1% cyclopentolate) was also analyzed in follow-up at 15 months of corrected age using the Retinomax 3 apparatus. According to the recommendations of the American Academy of Ophthalmology, refractive errors were defined as: high myopia (≥−6 Dsphr.), moderate myopia (from −3 to −6 Dsphr.), low myopia (from −0.5 to −3 Dsphr.), low (+0.5 to +3 Dsphr.), moderate (+3 to +6 Dsphr.) and high (≥+6 D Dsphr.) hyperopia, low (0.75 to 3 Dcyl.) and high astigmatism (>3 Dcyl.) [16]. One-was analysis of variance (ANOVA) was used for comparative analyses (statistics) of selected characteristics. Welch’s correction and non-parametric Kruskal-Wallis tests were used for large inequalities of variance. The CHI-squared test of independence was used to assess the effect of group on the ROP characteristic studied.

## 3. Results

The mean birth age in the analysed group was 25.8 ± 1.5 Hbd and the birth weight was 796.5 ± 166.1 g. The mean Apgar score was 4.62 ± 1.88 (range 1 to 8). The majority of infants came from single pregnancy (73%) and were born by Caesarean delivery (70%), which was most frequently performed due to life-threatening foetal distress and premature rupture of the foetal bladder. All subjects had anaemia requiring frequent blood transfusions—mean 4.89 ± 1.82 (ANOVA *p* = 0.0135). Significant differences occurred between groups 4 and 5 (*p* = 0.0245). Significant differences in red blood cells (RBC; mean 2.46 T/L ± 0.27) deficiency were observed between groups 3 and 5 (*p* = 0.0480); significantly lower Fe levels (64.47 µg/dL; ANOVA *p* = 0.003, statistically significant differences between groups 1 and 3 *p* = 0.0389; between groups 1 and 4 *p* = 0.0012 and between groups 1 and 5 *p* = 0.0094); mean Hbd was 4.71 mmol/L ± 0.87; average levels of platelets 86,600 G/L ± 6.37 were reduced in the five study groups (ANOVA *p* = 0.0085), a significant difference was observed in groups 2 and 4 (*p* = 0.0140) and between groups 2 and 5 (*p* = 0.0426). Other evaluated parameters were not statistically significantly different between the groups (Ht 25.1 ± 0.64%; TSH 2.67 ± 1.17 uIU/mL).

Comorbidities are presented in the table (Table 2). All infants required mechanical ventilation for 45 ± 18 days on average in different modes: SNIPPV, SIMV, VIVE, VG, HFV, HFNC, HFO. In twenty of the analysed eyes (n = 22; 63%), A-ROP with symptom plus in zone 1 was confirmed on ophthalmic examination using the RETCAM 3. In the remaining 11 eyes (n = 11; 35%) stage 3 of ROP with associated symptom plus (1, 2, 3), which required an individually matched treatment strategy (for the ROP/A-ROP groups, chi-squared test 7.676768 df = 4 *p* = 0. 10,416/9.330493 df = 4 *p* = 0.05335).

The first therapeutic intervention in groups 4 and 5 was performed the earliest—in the 33rd week of postconceptional age (PMA), whereas in the remaining groups of infants in 34.12 ± 2.0 (PMA), the mean follow-up time after the procedures was 21.44 ± 8.69 months.

**Figure 1:**
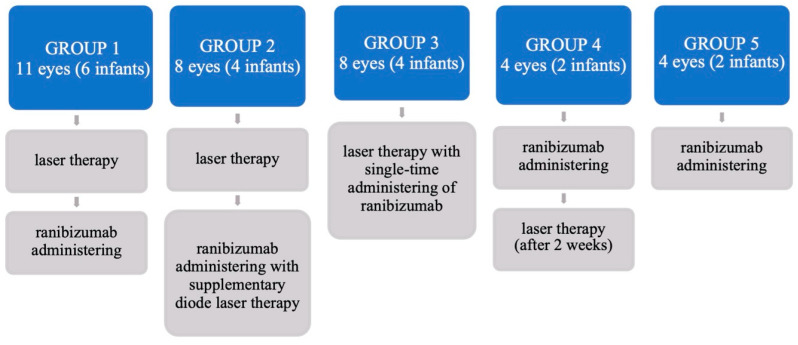
Sequence of therapeutic procedures used in the study groups with ROP.

The first group analysed consisted of 11 eyes in 6 infants (n = 11; Group 1)—Figure 1, in which the initial treatment was retinal diode-laser therapy (IRIDEX IQ 810), followed by intravitreal ranibizumab in the absence of a beneficial effect of the therapy, after a mean follow-up of 11.33 ± 5.91 days. The second group (n = 8; Group 2) comprised 8 eyes in 4 infants who received intravitreally Ranibizumab (IVR) with complementary diode-laser therapy at 10.67 ± 3.09 days after the first laser course. The third group included 8 eyes in 4 premature infants (n = 8; Group 3), in whom laser-therapy with simultaneous intravitreal administration of Ranibizumab (Lucentis) was performed. Subsequently, in 4 eyes in two premature infants (n = 4; Group 4), due to non-transparent optic centers, Ranibizumab intravitreal injection was initially applied and laser therapy was performed after 2 weeks. The last group (n = 2; Group 5), consisted of 4 eyes in two infants with A-ROP who were treated with Ranibizumab intravitreal administration only. A lower dose of Ranibizumab, i.e., was administered intravitreally in 12 eyes in 6 infants (n = 12); including two eyes from Groups 1 and 4, four eyes from Groups 3 and 5—Figure 1.

Three patients in Group 1 and two patients in Group 4 required additional laser therapy, which was performed after an average of 11.6 ± 12.86 days of observations. In the studied groups of premature infants, five of them did not attend follow-up visits and were from other cities, while one infant died outside the hospital due to sudden infant death syndrome (SIDS). One may wonder if it was not a vascular complication after IVR administration. In another extremely low-weight premature infant from Group 3, initially treated with laser therapy and intravitreal Ranibizumab with favourable results, the next outpatient follow-up performed 2 months after the start of the therapy revealed bilateral retinal detachment, which was unsuccessfully treated on the153rd day with posterior vitrectomy. A careful assessment of the risk factors for these complications indicated that these causes could have been either a bacterial inflammatory infection or a urinary tract inflammation which occurred 10 days after the completed ophthalmological treatment (simultaneous administration of Ranibizumab and diode-laser) of still clinically active severe ROP or BCG vaccination performed in outpatient care 28 days after the combined ophthalmological treatment, i.e., still in the course of healing of active ROP (infants’s 97th day of life). In this case, one may also wonder whether the recurrence of progressive ROP was not related to complications in the course of a severe bacterial infection complicating ROP.

Regression of ROP with beneficial retinal attachment, with the absence of vascular, retinal and intravitreal complications, was observed in the remaining described group of 13 infants (92%). No recurrence of secondary ROP was noted during the subsequent systematic two-year follow-up of these patients. The refraction was examined in 11 premature infants (Retinomax) and refractive defect was recorded in values ranging from −7.5 to +4.5 of spherical dioptres (Dsphr.). In the group of 5 preterm infants (45%), myopia was found with values from −0.75 to −7.5 Dsphr.; three of them were infants from Group 1: one infant from Group 2 and one from Group 3. Positive medical history with myopia of about −1.0 Dsphr. was noted in two of the infants mentioned above. In the remaining group of infants, mild hyperopia was noted, which is normal refraction in this age group (+0.5 to +4.5 Dsphr.; mean +2.5 Dsphr. (±1.5). In the ANOVA test, differences in spherical refraction were statistically significant between Groups 3 and 4 (*p* = 0.0005) and between Groups 3 and 5 (*p* = 0.0024). Whereas in cylindrical refraction, a statistically significant difference was shown between Groups 2 and 4 (*p* = 0.0135); in Kruskal-Wallis tests.

In the following 24 months of follow-ups, neurological and transcranial ultrasonography examinations did not confirm any worsening of the follow-up ophthalmic results or any changes in the premature’s motor development after monotherapy 0.12 mg IVR or diode-laser therapy as well as combined treatment in the course of severe forms of ROP.

## 4. Discussion

Early detection of retinopathy of prematurity as well as appropriate planning and implementation of treatment are crucial for the long-term beneficial effects of vision in low- and extremely low-weight premature infants. Screening of premature infants plays an important role in the diagnosis of ROP and in case of active 3 ROP/A-ROP and plus 2,3 ROP, or 3 ROP plus; it is important to urgently initiate the appropriate treatment strategy: IVR monotherapy or diode-laser as well as combined treatment i.e., anti-VEGF intravitreal injection IVR together with diode laser retinal panphotocoagulation.

Standard treatment of ROP, diode-laser photocoagulation inhibits VEGF production by destroying the avascularised, peripheral, ischemic retina, which produces various growth factors such as VEGF, transforming growth factor, beta_3_ TGF-β_3_), erythropoetine (EPO) [8], angiopoietin-like protein 4, ANGPTL4 [9] and others, but does not affect the level of VEGF, which has already been secreted into the vitreous body and subretinal area. Moreover, failure to inhibit the secretion of these substances causes a number of complications, leading to retinal detachment that may result in visual impairment or even blindness. Use of diode-laser therapy remains the main strategy for ROP treatment, but it is known that laser-coagulation can cause complications in the form of loss of the peripheral visual field, cataracts, secondary glaucoma, optic nerve atrophy, macular ectopia, pit pigmentation abnormalities [17], choroidal and retinal haemorrhage or haemorrhage into the vitreous [18] with corneal oedema, posterior adhesions, pupillary membranes, anterior chamber plaque and haemorrhage [19].

Importantly, in individual cases of severe bronchopulmonary dysplasia, it is possible to administer an anti-VEGF drug (IVR) for general anesthesia [20,21].

It has also been proved that anti-VEGF treatment with Ranibizumab causes stable retinal vascularisation with low rates of complications and recurrence of ROP [22]. However, isolated cases of retinal detachment [23,24] and intraocular inflammation after anty-VEGF injections are known in the literature [25,26].

Differences in the percentage of beneficial effects after treatment with Ranibizumab remain disputable, which is why it is so important to present one’s own experience regarding the benefits of the therapy, especially when using lower than recommended doses of anti-VEGF. There is little data on the association between myopia and intravitreal Ranibizumab, and most of the papers refer to Bevacizumab. Results of the study by Kimyon et al. indicate a higher incidence of myopia with high myopia in the group of premature infants treated with Bevacizumab compared to Ranibizumab [22,27]. Intravitreal Bevacizumab (IVB) was compared to Ranibizumab in an ophthalmic study at corrected 1 year of age (14.6% versus 0.0% IVR), as well as at 3 years of age (16.7% versus 0.0%) [28]. In contrast, another study found no difference regarding myopia between the two groups at a corrected age of 3 years. However, unlike Ranibizumab, patients receiving Bevacizumab had a shallower anterior chamber and were more likely to have ocular non-marring, or anisometropia, observed [29]. Compared to laser treatment for ROP in zone I, a higher incidence of myopia, including high myopia, was observed in infants with diode-laser retinal photocoagulation than in premature treated with anty-VEGF solutions [30]. Another study found that high-grade myopia occurred only in 2.7% of patients in the Bevacizumab-treated premature group, compared with 42% in the laser-treated nowborns [20].

However, in our observations, myopia from −0.75 to −7.5 D was noted in 5 prematures, which is 45% of the studied group and higher than in the quoted studies. These were infants in whom the first stage of treatment procedure was diode-laser only one or laser combined with intravitreal injection of Ranibizumab. In contrast, in the group where Ranibizumab was the first or only one treatment, no myopia was observed. Authors of this report observed, that myopic spherical refraction was correlated with BW (*p* = 0.048 vs. *p* = 0.845) or GA (*p* = 0.01 vs. *p* = 0.21) compared to premature infants without ROP. Moreover, it may be connected with the others than considered risk factors. It is interesting that the parents of the analyzed myopic children are also myopic, but this is a topic for further consideration [31].

Several studies presented in the literature have demonstrated systemic suppression of VEGF and other growth factors after intravitreal administration of anti-VEGF in premature infants [32,33], which may raise concerns about the occurrence of future late or long-term complications in the form of mental and/or motor developmental delay along with abnormal neurological development. After intravitreal administration of Ranibizumab, there is barely detectable or no suppression of serum VEGF compared to Bevacizumab, where significant suppression of serum VEGF is observed [34]. Moreover, VEGF suppression in this group of babies was longer than in adults, and Ranibizumab, despite a sharp decline one day after injection, had plasma-VEGF levels returned to baseline after one week, indicating that Ranibizumab does not induce prolonged systemic suppression of VEGF. This stands in contrast to Bevacizumab, where the serum level was significantly reduced for about 2 months [35].

This may result in ROP recurrence and consideration of more frequent follow-up visits for already treated infants. Similarly, Hoerster et al. showed that ranibizumab reduces VEGF levels for about 2–3 weeks and then returns to normal values about 4 weeks after starting treatment [30]. The stabilization of the clinical condition and the lack of ROP recurrence in the 24-month follow-up are also reported by the authors of this article, which is consistent with the observations of Hoerster et al.

With regard to the lower doses of anti-VEGF used so far, it should be mentioned that single studies comparing the efficacy of the significantly lower dose of Ranibizumab commonly used to date, are already available in the literature. Furthermore, the results of these studies indicate that the 0.12 mg dose, representing 24% of the standard adult dose is as effective as the 0.20 mg dose, representing 40% of the adult dose, which suggests that the lower dose can be administered in severe ROP/A-ROP with equally beneficial effects and efficacy [15]. The authors of this paper also emphasise the beneficial effect of treatment after lower doses of Ranibizumab in a group of 8 eyes (26%) with normal retinal vasculature development in retinal zone 2–3 in infants treated with monotherapy, indicating that an accurate and prompt diagnosis makes it possible to start treatment with the lowest doses of anti-VEGF substances, which is undoubtedly of great importance for an extremely low-weight infant in terms of reducing side effects in their neurological development and pulmonary status for the future. Similarly, RAINBOW tests demonstrated the efficacy of 0.1 mg of ranibizumab in 75% of infants, compared to 80% of infants who received the drug at 0.2 mg; these results were higher compared to the laser therapy group (66%) [11]. It is worth noting that the use of Ranibizumab, due to its shorter half-life and potentially lower systemic impact, may be a safer therapeutic option for the treatment of ROP, which was also noted in our observations.

It should be emphasised that the feature differentiating anti-VEGF from diode-laser therapies is the need for a much longer period of observation of the retinal clinical condition. According to Lyu et al., observation for recurrence of ROP should last until complete regression of ROP duration with zone 3 vascularisation and should then be individualised to the patient, although it should be significantly more frequent in the first 12 weeks after therapy with ranibizumab compared to laser therapy. In this authors’ study, recurrence of ROP occurred in up to 64% of eyes at a mean of 7.9 ± 2.7 weeks after IVR [36]. Similarly to Zhou’s study, ROP recurrence after Ranibizumab occurred at 7.3 ± 3.5 weeks during 21 weeks of observations [24]. Another study by Wong et al. shows that all ten eyes that received anti-VEGF treatment showed initial regression of ROP. However, reactivation of ROP occurred in 5/6 (83%) eyes with Ranibizumab, an average of 5.9 weeks after the start of treatment [37]. In comparison, in the BEAT-ROP test, the mean time from diode laser initiation to ROP reactivation was 6.2 ± 5.7 weeks and 16 ± 4.6 weeks after Bevacizumab inclusion. Earlier reactivation of ROP after IVR monotherapy compared to IVB may be related to the shorter half-life in the vitreous body of Ranibizumab compared to Bevacizumab. The authors of this article point out that in the study group the complication of retinal detachment in both eyes was observed only in 1 infant (2 eyes), in whom the simultaneous administration of ranibizumab with laser therapy was used, which constitutes 6.9% in the group of infants treated with combined therapy; however, the authors did not conduct ophthalmological follow-up after the end of hospitalization (the infant did not come for follow-up examination). We treat this complication as a recurrence of ROP in the course of the risk factor, which was urinary and bronchial tracts infection with positive bacterial culture.

A lower percentage of complications in the treatment of ROP than the aforementioned results in the group presented by us may be due to the proper qualification for the performance of laser procedures or intravitreal injections of anti-VEGF into the blood vessels by one and the same experienced ophthalmologist, with observance of frequent appointments for individualised control examinations (every 2–3 days or successively during the first 2 weeks, then every 5–7 days until the infant is discharged from hospital; then successively every 2 weeks until the infant is 6 months old and successively every 2 months until the infant is 2 years old, with short intervals in between).

There are numerous studies in the world literature which compare the efficacy of both of the above mentioned methods: monotherapy or combined treatment [21,38,39]. The studies by Hu et al. show that intravitreal injection of Ranibizumab combined with laser therapy can be effective in severe A-ROP, which is also observed in our study, where the efficacy of combined treatment for simultaneous A-ROP is 89%, and for ROP3+ even 100% [40,41]. In some severe cases with coexisting systemic conditions, including inflammatory bacterial or fungal diseases, monotherapy with only Ranibizumab may not be sufficient to treat A-ROP. This is also evidenced by our results, where in 5 eyes in 3 infants (67%) there were infections with a confirmed aerobic and anaerobic bacterial, fungal or CMV pathogen: bacterial meningitis, complicating fungal sepsis, necrotizing enterocolitis, pneumonia combined with bronchopulmonary dysplasia, respiratory distress (thus low oxygen saturation and apnea), with coexisting anaemia, IVH, there was a need for combined treatment and addition of laser therapy to achieve beneficial efficacy. A generally known opinion is that Ranibizumab is effective as initial therapy in selected cases of A-ROP not complicated by additional systemic infection. In the remaining majority of cases of advanced A-ROP with features of choroidal neovascularisation and features of retinal hypoxia and ischaemia and severe ROP+, it is necessary to support (support? How?) this therapy and to continue treatment with diode laser [42]. Similar findings can be found in Gotz-Więckowska’s study, where patients with active plus ROP, referred to treatment at a reference clinical centre late, who are at high risk of retinal detachment, can be treated with a two-stage treatment—first with laser photocoagulation and then with ranibizumab injection. In these patients, simultaneous diode-laser therapy and intravitreal injection may be the most beneficial [43]. According to our results, it is important to emphasise the necessity of considerable individualisation of therapy in order to obtain a satisfactory treatment result. Patients in group 3 in our observations, due to the severity of the ROP process, were treated with combined diode laser therapy with simultaneous administration of ranibizumab, similarly to patients in groups 1 and 2 in whom anti-VEGF was administered 9.75 ± 3.63 days after laser therapy. The simultaneous performance of two therapeutic procedures is also less burdensome for the infant, because it eliminates the need for another general anaesthesia. Data in the literature indicate that another retrospective study on the use of intravitreal anti-VEGF in the treatment of ROP type 1 also (after initial regression) showed a higher recurrence rate with Ranibizumab (40%) compared with Bevacizumab (10%) [44]. Our observations remain contradictory to the aforementioned finding, as in severe ROP after using both Ranibizumab monotherapy and Ranibizumab combine treatment, ROP recurrence was reported in only 6.5% of cases during almost 2 years of follow-up (21.44 ± 8.7 months). According to the authors’ observation, it seems that an important element determining the beneficial effect of treatment is the early detection of discrete retinal vascular or vitreous changes in the early stages of the development of the disease, or changes in echogenicity in the posterior vitreous detected by B ultrasound, which at this stage already indicates the activation and development of the disease, and the effect of rapid therapeutic intervention with properly selected therapy results in beneficial treatment results, which is possible with extensive experience of the examiner.

It is also important to determine the order in which therapies are implemented when they are combined. Due to the still existing controversies regarding the use of different forms of anti-VEGF treatment, so far there is no single standard scheme of conduct for the combined treatment of ROP in the world literature. Our observations have shown that there is no significant relationship between the sequence of therapeutic interventions. Treatment effects were favourable both when laser therapy was performed with Ranibizumab administration simultaneously (ROP3 and plus disease) and when therapy was performed with laser therapy as the first treatment step and successive anti-VEGF administration at different time distances, in our case it was 11.11 ± 5.15 days after the initial laser therapy. These results differ from the study carried out by Gotz-Więckowska et al. where the mean time between interventions was longer and was 19 days [38]. A favourable effect was also obtained in infants with A-ROP, where the first treatment step was intravitreal administration of Ranibizumab, followed by laser therapy after 13.5 ± 0.5 days on average. Another retrospective study by Arámbulo et al. showed that in the group treated with Ranibizumab and laser therapy, favourable results were obtained in 70.7% of eyes and more favourable results were observed when patients received an initial IVR injection and follow-up laser treatment after 3 weeks (100% positive results) than in patients who were initially treated with laser photocoagulation followed by IVR (57.1% positive results) [45]. Different results were obtained in our groups of premature infants. In the group initially treated with IVR injection followed by laser photocoagulation (Group 1), additional laser therapy was required in 5 eyes. In all infants with severe 3 ROP (Group 4) treated consecutively in the first stage with IVR and then with supplementary laser therapy, a second laser therapy was necessary. Finally, the beneficial effect of ROP treatment in this group was obtained in 100% of cases. In the case of monotherapy with a dose of 0.25 mg ranibizumab in a group of 87.5% of infants in the study by Aldebasi et al., positive results were obtained in terms of regression of neovascularisation [46]. In the selected cases presented by us using monotherapy, lesion regression was achieved at a dose of 0.12 mg, which may indicate that proper diagnosis and classification of ROP and immediate initiation of anti-VEGF therapy may prove to be effective, without the additional inclusion of laser coagulation, usually in neonates with fewer systemic burdens. Our observations indicate that laser therapy remains a widely accepted standard of treatment, where the effects of this therapy are known in the literature in the form of long-standing and long-term data with a level of evidence so far unprecedented in studies regarding anti-VEGF treatment.

Another interesting aspect is the fact of the predominance of male infants in our group (87.5%). Similar to the study of Xu [40], where 80% of the patients were boys, which may suggest that they show a predilection to develop severe forms of ROP. However, due to the small size of the study groups, further studies are required to confirm this hypothesis.

### Limitations

The limitation of our study was a small group of premature infants treated only with monotherapy. Of course, we take into account summaries of treatment effects and refractive indexes after a longer period of observation of the same groups of children undergoing ROP therapy. Another aspect of interest is the effect of ROP treatment obtained based on the order of the procedures used—anti-VEGF, laser or laser-anti-VEGF. The gathering of information on treatment in these groups of premature babies is important for ophthalmologists and neonatologists. Therefore, selection of larger groups of patients becomes become the ultimate goal.

## 5. Conclusions

It seems that the moment of early therapeutic intervention, combined with the selection of the appropriate procedure for the treatment of severe forms of ROP is the most important element of the effective results of therapy. Regardless of the sequence of therapeutic procedures undertaken in severe ROP, treatment rarely ends with monotherapy alone, and the effects of combined therapy conducted in parallel are more favorable, without long-term complications in our two-year observations. The results of our observations indicate that in selected cases, a lower dose of ranibizumab, 0.12 mg, used in our treatment regimens as monotherapy in A-ROP or in combination therapy in ROP 3(+), resulted in beneficial treatment effects and did not increase the risk of ROP complications and recurrences.

It seems that individualization of therapy and early therapeutic intervention, combined with the selection of appropriate management in the treatment of severe forms of ROP, is the most important element of effective therapy results. Irrespective of the chosen form of treatment, it seems that in A-ROP without a feature plus the first administration of IVR may bring improvement even when it requires another administration of IVR in monotherapy. First administration of IVR in combination therapy may reduce the occurrence of myopia in the future. Simultaneous diode-laser and IVR procedures should be considered for severe ROP with a plus feature with an abrupt and progressive course was a need for combined treatment and addition of laser therapy to achieve beneficial efficacy.

The authors of this article uphold the opinion of other authors that in the case of concomitant infectious multi-organ disorders in severe ROP, combined therapies should be used depending on the stages: in A-ROP(+), IVR and then laser or in 3 ROP(+), laser and IVR, respectively.

The results of our observations indicate that the lower dose of Ranibizumab, 0.12 mg, used in our treatment regimens, both in monotherapy in A-ROP and ROP 3(+) or in combined therapy, brought beneficial treatment effects without the risk of ROP recurrence in the 2-year follow-up. The authors do not rule out the risk of vascular or pulmonary neurological complications in the short and long term, even with a dose of 0.12 mg IVR. Early and targeted ophthalmological intervention can prevent further development of the disease, lead to its inhibition and create safe conditions for the proper development of the eye.

## Figures and Tables

**Table 1:** Frequency of comorbidities in the studied groups of premature infants.

Comorbidities	Frequency of Comorbidities in the Study Group (%)
Respiratory distress syndrome (RDS)	72%
Bronchopulmonary dysplasia (BPD)	78%
Pneumonia	61%
Patent Foramen Ovale (PFO)	56%
Intraventricular Haemorrhages (IVH) 3rd and 4th degree	44%
Necrotizing Enterocolitis (NEC)	28%
Patent Ductus Arteriosus (PDA)	33%
Meningitis	28%
Hypothyreotoxcosis	22%

Comorbidities—coexisting diseases that occurred in premature infants expressed in percentages.

**Table 2:** Clinical characteristics of 18 extremely low-weight premature infants included in the ophthalmological study.

No.	Sex	GA	Weight	ROP	Plus Symptom	PMA Laser	PMA IVR	Time between Procedures	2. Laser (Number of Days from the Last Procedure)	Therapy Effects	
1	M	25	800	A-ROP	plus	34	36	9		regression	
2	M	27	950	A-ROP	plus	35	35.5	3		regression	
3	M	26.5	750	3B	plus	34	35.5	10	3	regression	GROUP 1: Laser →IVR
4	M	24	750	A-ROP	plus	32	34	15	3	regression	
5	M	27	990	3B	plus	39.5	41	9	7	regression	
6	F	25	600	3B	plus	35	38	22		regression	
**No.**	**Sex**	**GA**	**Weight**	**ROP**	**Plus symptom**	**PMA laser**	**PMA IVR + laser**	**Time between procedures**		**Therapy effects**	
1	F	24	590	* A-ROP	plus	33	34	8		regression	
2	M	28	1000	3B	plus	36	40	15		regression	GROUP 2: Laser → laser + IVR
3	M	24	700	* A-ROP	plus	33.5	35	9		regression	
4	M	27	670	3	plus	36	38	14		regression	
**No.**	**Sex**	**GA**	**Weight**	**ROP**	**Plus symptom**	**PMA laser + IVR**				**Therapy effects**	
1	F	26	870	3B	plus	36.5				regression	GROUP 3: Laser + IVR
2	M	23.5	860	* A-ROP	plus	33				complication	
3	M	27	900	3B	plus	35				regression	
4	M	29	970	* A-ROP	plus	35				regression	
**No.**	**Sex**	**GA**	**Weight**	**ROP**	**Plus symptom**	**PMA IVR**	**PMA laser**	**Time between procedures**	**2. laser (number of days from the last procedure)**	**Therapy effects**	
1	M	25	400	* A-ROP	plus	33	35	13	7	regression	GROUP 4: IVR →laser
2	M	25	800	* A-ROP	plus	32	34	14	8	regression	
**No.**	**Sex**	**GA**	**Weight**	**ROP**	**Plus symptom**	**PMA** **IVR**				**Therapy effects**	
1	M	25	700	A-ROP	0	35				regression	GROUP 5: IVR
2	M	26	940	A-ROP	0	33				regression	

Abbreviations: bold indicates a feature—or parameter that is characterized in the table. No—the ordinal number of the baby; GA—gestational age; ROP—retinopathy of prematurity; IVR—Ranibizumab administered intravitreally; laser + IVR—simultaneous performance of diode laser panphotocoagulation and administration of Ranibizumab intravitreally; A-ROP—Aggressive ROP; * A-ROP—Aggressive ROP with an advanced symptom plus, the course of which was rapid, severe and required the addition of anti-VEGF; Plus-symptom—the plus clinical characteristic feature of 2 or 3 ROP and A-ROP indicating active ROP; PMA Lucentis—postconceptional age while the Lucentis intravitreal injection was performed; PMA laser—postconceptional age, while the laser-diode procedure was been made; regression—withdrawal of vascular and retinal symptoms in 3 ROP(+)—with vascularization of the previously avascular area in A-ROP—complication.

## Data Availability

The presented data are available in the patient’s medical records at the ophthalmology center where the patient was examined.
